# A comprehensive analysis of germline predisposition to early-onset ovarian cancer

**DOI:** 10.1038/s41598-024-66324-2

**Published:** 2024-07-13

**Authors:** Klara Horackova, Petra Zemankova, Petr Nehasil, Michal Vocka, Milena Hovhannisyan, Katerina Matejkova, Marketa Janatova, Marta Cerna, Petra Kleiblova, Sandra Jelinkova, Barbora Stastna, Pavel Just, Tatana Dolezalova, Barbora Nemcova, Marketa Urbanova, Monika Koudova, Jana Hazova, Eva Machackova, Lenka Foretova, Viktor Stranecky, Michal Zikan, Zdenek Kleibl, Jana Soukupova

**Affiliations:** 1 Institute of Medical Biochemistry and Laboratory Diagnostics, First Faculty of Medicine, Charles University and General University Hospital in Prague, Prague, Czech Republic; 2 Institute of Pathological Physiology, First Faculty of Medicine, Charles University, Prague, Czech Republic; 3 Department of Paediatrics and Inherited Metabolic Disorders, First Faculty of Medicine, Charles University and General University Hospital in Prague, Prague, Czech Republic; 4 Department of Oncology, First Faculty of Medicine, Charles University and General University Hospital in Prague, Prague, Czech Republic; 5 Department of Genetics and Microbiology, Faculty of Science, Charles University in Prague, Prague, Czech Republic; 6 Institute of Biology and Medical Genetics, First Faculty of Medicine, Charles University and General University Hospital in Prague, Prague, Czech Republic; 7 Department of Biochemistry, Faculty of Science, Charles University, Prague, Czech Republic; 8 Centre for Medical Genetics and Reproductive Medicine, GENNET, Prague, Czech Republic; 9 Department of Cancer Epidemiology and Genetics, Masaryk Memorial Cancer Institute, Brno, Czech Republic; 10 Department of Gynecology and Obstetrics, Bulovka University Hospital and First Faculty of Medicine, Charles University, Prague, Czech Republic

**Keywords:** Ovarian cancer, Early-onset, Germline whole exome sequencing, Polygenic risk score, HLA, Mutation burden, Gynaecological cancer, Cancer genetics

## Abstract

The subset of ovarian cancer (OC) diagnosed ≤ 30yo represents a distinct subgroup exhibiting disparities from late-onset OC in many aspects, including indefinite germline cancer predisposition. We performed DNA/RNA-WES with HLA-typing, PRS assessment and survival analysis in 123 early-onset OC-patients compared to histology/stage-matched late-onset and unselected OC-patients, and population-matched controls. Only 6/123(4.9%) early-onset OC-patients carried a germline pathogenic variant (GPV) in high-penetrance OC-predisposition genes. Nevertheless, our comprehensive germline analysis of early-onset OC-patients revealed two divergent trajectories of potential germline susceptibility. Firstly, overrepresentation analysis highlighted a connection to breast cancer (BC) that was supported by the *CHEK2* GPV enrichment in early-onset OC(*p* = 1.2 × 10^–4^), and the presumably BC-specific PRS_313_, which successfully stratified early-onset OC-patients from controls(*p* = 0.03). The second avenue pointed towards the impaired immune response, indicated by *LY75-CD302* GPV(*p* = 8.3 × 10^–4^) and diminished HLA diversity compared with controls(*p* = 3 × 10^–7^). Furthermore, we found a significantly higher overall GPV burden in early-onset OC-patients compared to controls(*p* = 3.8 × 10^–4^). The genetic predisposition to early-onset OC appears to be a heterogeneous and complex process that goes beyond the traditional Mendelian monogenic understanding of hereditary cancer predisposition, with a significant role of the immune system. We speculate that rather a cumulative overall GPV burden than specific GPV may potentially increase OC risk, concomitantly with reduced HLA diversity.

## Introduction

Ovarian cancer (OC; including tumors of ovary, fallopian tube and peritoneum) remains the deadliest gynecological malignancy ^1^. OC shows variability not only in anatomy but also at the cellular and molecular levels, including different histological types. Majority of the OC patients are diagnosed with epithelial, particularly high-grade serous carcinoma (HGSC) at advanced stages associated with a poor prognosis ^2,3^. The lifetime OC risk is about 1.3%^1,4^. Nevertheless, there is a number of known factors, including genetic predisposition, that may substantially modify the OC risk. Germline pathogenic variants (GPV) in OC predisposition genes including the most frequently affected genes *BRCA1/BRCA2* are of the highest impact. Identification of the GPV allows to stratify women according to the OC risk and, subsequently, to offer the carriers appropriate surveillance management including targeted therapy. The proportion of hereditary OC associated with GPV in homologous recombination and mismatch repair pathway genes is reported to be around 25%; however, it varies among OC histological subtypes and is the highest in HGSC^5,6^. OC rates are the highest in women aged 55–64 years with a median age of diagnosis at 63 years ^1^. In contrast, OC in young women (≤ 30 years) is rare accounting for less than 5% of all OC cases^1^.

Understanding the genetic basis of early-onset OC is crucial for unraveling the complex factors that contribute to its onset and progression. In many solid malignancies, early onset is a hallmark of hereditary predisposition. However, the proportion of early-onset OC attributable to GPV in OC predisposition genes is uncertain with only a few performed studies analyzing a limited number of early-onset OC patients diagnosed before 30 years of age. Interestingly, all these studies identified no or very low frequency of GPV in OC predisposition genes^6–11^. Despite the limited number of analyzed early-onset OC patients, the lack of GPV in OC predisposition genes, particularly *BRCA1/BRCA2*, cannot be simply explained by a different representation of individual histological OC subtypes. Other mechanisms, including polygenic inheritance and immune response, might be involved in pathogenesis of early-onset OC. Polygenic inheritance resulting from the combination of multiple alleles with low impact on OC risk could contribute to early-onset OC development. Several sets of these low-penetrance alleles for stratification of individuals according to their OC risk have been published^12–21^. In addition, certain HLA genotypes have been previously associated with increased susceptibility to various diseases including early-onset OC; tumor neoantigen recognition might also depend on genetic variability in HLA regions^22^. Thus, early-onset OC represents a unique and compelling area of investigation within the broader landscape of ovarian malignancies.

In our study, we aimed to comprehensively characterize germline genetic landscape of early-onset OC in a set of 123 patients diagnosed before the age of 30 years using DNA/RNA whole exome sequencing (WES) including HLA analysis and polygenic risk score (PRS) analysis. In addition, we analyzed the genotype data in the clinical and histopathological context and in comparison, with population-matched histology/stage-matched late-onset OC patients and non-cancer controls.

## Patients and methods

### Patients and controls

We enrolled 123 patients diagnosed with early-onset (< 30 years) OC (denoted herein as “early-onset OC”). Peripheral-blood derived gDNA of 123 patients and peripheral-blood derived total RNA (available in 71 patients) were analyzed using WES. Mean age at OC diagnosis was 25.4 years (15–30), 93 patients were diagnosed with invasive ovarian tumors (61 epithelial, 15 non-epithelial denoted herein as “other”, and 17 with unspecified histology) and 30 with borderline tumors of ovary (BTO) (Table 1; Supplementary Table S1).

**Table 1:** Characteristics of 123 early-onset OC patients.

Histology	N (%)
Epithelial OC	61 (49.6)
Type I	46 (37.4)
- LGSC	22 (17.9)
- Mucinous	15 (12.2)
- Endometrioid	6 (4.9)
- Clear cell	3 (2.4)
Serous NOS	11 (8.9)
Type II	4 (3.2)
- HGSC	2 (1.6)
- Undifferentiated	2 (1.6)
NA	17 (13.8)
BTO	30 (24.4)
Other	15 (12.2)

Multiple primary tumors	
No	110 (89.4)
Yes	13 (10.6)

Family cancer history	
Positive	88 (71.6)
- Early onset cancer < 40y in family history	24 (19.5)
- Hematological malignancy in family history	15 (12.2)
Negative	33 (26.8)
NA	2 (1.6)

BTO, Borderline Tumors of Ovary; LGSC, Low-Grade Serous Carcinoma; HGSC, High-Grade Serous Carcinoma; NA, not available; NOS, not otherwise specified; OC, Ovarian Cancer.

For PRS and survival analysis, we employed additional OC patients analyzed previously ^6^ (details in Methods; Supplementary Table S4).

We employed several sets of population-matched controls:

- *for DNA variant prioritization*: 227 healthy females older than 60 years with no personal or first-degree family cancer history (denoted herein as “super-controls”).
- *for RNA splicing event prioritization*: 61 unselected non-cancer females with available RNA WES data (denoted herein as “non-cancer controls”).
- *for risk calculation in case–control analysis*: 378 unselected individuals provided by the National Center for Medical Genomics^23^ (accessed on 3/2023; denoted herein as “unselected controls”).
- *for HLA analysis*: “super-controls” and a second group of 5099 unselected individuals (data for HLA-C and HLA-DQB1 were available for 4669 and 4049 individuals, respectively) from Czech National Marrow Donors Registry ^24^ (accessed on 11/2023; denoted herein as “HLA controls”).
- *for PRS analysis*: 1403 non-cancer females negative for GPV in hereditary breast, ovarian and pancreatic (HBOP) cancer predisposition genes^25^ (denoted herein as “PRS controls”)^26,27^.

All patients and controls were Caucasians of Czech origin. Written informed consent was obtained from all patients and controls. The study was approved by the Ethics Committee of the General University Hospital in Prague and performed in accordance with the Declaration of Helsinki.

### NGS library preparation

Sequencing libraries were prepared as described previously^28–30^ with minor modifications (detailed in Supplementary Methods) and targeted whole exome (KAPA HyperExome panel, Roche; capture target 43 Mb) and 843 SNP including 65 SNP previously reported to associate with OC PRS (custom HyperChoice panel, Roche; Supplementary Table S2)^26,27^. The minimal mean coverage was 30 × for DNA WES, 200 × for RNA WES (with coverage of exon 11 in BRCA1 mRNA as a coverage quality marker) and 20 × for PRS genotyping analysis.

### Bioinformatics pipeline for variant analysis

The fastq data from DNA WES were analyzed as described previously^28^ with minor modifications (detailed in Supplementary Methods). Copy number variations (CNV) were analyzed using CNVkit as described previously^28^. The fastq data from RNA WES were mapped to hg19 using STAR aligner to generate BAM files^31^. Subsequently, duplicates were removed using Picard tools v1.129^32^ and analyzed by regtools^33^ and SCANVIS^34^.

### Variant filtration and prioritization for the burden analysis

Variant prioritization (considering population frequency or sequencing quality) and variant classification were performed independently for DNA events (separately for substitutions/short-medium length indels and CNV analysis) and RNA events as described in Supplementary Methods. Final list of GPV (Supplementary Table S3) was used for the subsequent statistical analysis. The following genes were considered HBOP cancer predisposing: *ATM, BARD1, BRCA1, BRCA2, BRIP1, CDH1, CDKN2A, CHEK2, MLH1, MSH2/EPCAM, MSH6, NF1, PALB2, PMS2, PTEN, RAD51C/D, STK11,* and *TP53*^25^.

### Statistical analyses

Statistical analysis was performed in R v.4.2.0; paired and multiple comparisons were performed using Fisher exact and Kruskal–Wallis tests, respectively; *p* < 0.05 was considered significant. Gene burden analysis of the final list of GPV in patients and unselected controls was performed using Fisher exact test, the 3 × 10^−7^ exome-wide Bonferroni corrected *p*-value threshold was considered significant^35^. Overrepresentation analysis of the final list of GPV in patients was performed using online WEB-based GEne SeT AnaLysis Toolkit (www.webgestalt.org, accessed on 11/2023)^36^.

### Human leukocyte antigen (HLA) analysis

HLA analysis was performed from DNA WES data of 123 early-onset OC patients and 227 super-controls using SpecHLA tool genotyping of HLA-A, B, C, DPA1, DPB1, DQA1, DQB1, and DRB1^37^. The HLA genotypes were curated to the level of amino acid (four-digit resolution). The frequency of HLA alleles in early-onset OC and controls (HLA- and super-controls) were compared using Fisher test. Due to unavailability of HLA-DPA1, DPB1, and DQA1 genotypes and information about zygosity in HLA controls, the frequencies of the three genotypes and the zygosity status were statistically evaluated only to super-controls.

### PRS analysis

We performed genotyping of 11 SNP sets, including 10 OC^12–21^ and one breast cancer (BC)^38^, in 122 early-onset OC patients (PRS in one patient could not be assessed) and 85 histology- and stage-matched late-onset OC patients (aged > 40 years; denoted herein as “histology/stage-matched OC”); and 78 population-matched late-onset HGSC patients negative for GPV in high-penetrance OC predisposition genes (Supplementary Table S1, S2, S4).

Raw NGS data were processed by an in-house bioinformatics pipeline as described previously^28^. PRS was calculated as described by Borde et al.^39^ (Supplementary Methods). Differences between the standardized PRS values of patients and PRS controls were assessed in R v.4.2.0 using *t*-test.

### Survival analysis

The survival analysis was performed using the Kaplan–Meier analysis and the log-rank test in R v.4.2.0. Vital status was available in 82 early-onset (median 25.1 years; 14.8–30.8 years) and 917 previously analyzed, late-onset (> 30 years; median age 58.1 years; 31.2–91.8 years) OC patients^6^. In addition, subgroup analyses were performed using groups stratified by OC histology and by germline *BRCA1/BRCA2* (*gBRCA1/2*) status. Individual OC patients’ subgroups included into the survival analysis are summarized in Supplementary Table S1, S4, S6.

## Results

### DNA/RNA WES

**Figure 1:**
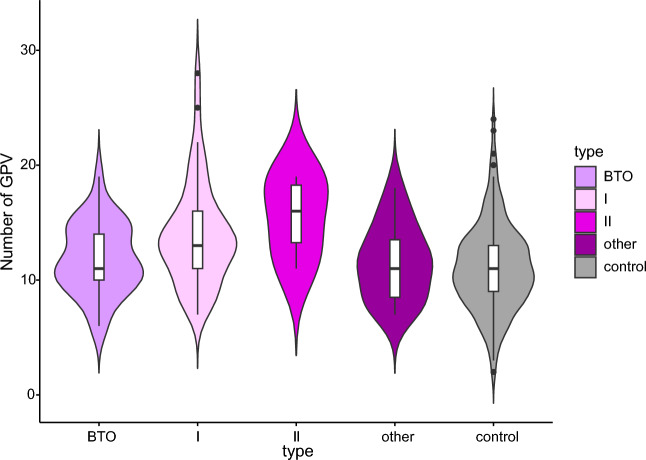
Overall germline pathogenic variant (GPV) burden in early-onset OC patients and super-controls (non-cancer individuals without family cancer history). *BTO, Borderline Tumors of Ovary.*

In our cohort of 123 early-onset OC patients, a total number of 1563 germline GPV (1506 unique variants) in 1390 genes stemmed from DNA (SNV and CNV accounting for 95.3% and 1% of GPV, respectively) and RNA WES (additional 3.7% of GPV). Median number of GPV per patient was 13 (ranged 2–28). The GPV mutation burden was significantly lower in super-controls compared to early-onset OC patients (*p* = 3.8 × 10^–4^) and tended to be lower in BTO and patients with other (non-eptihelial) OC compared to type I [low-grade serous (LGSC); endometrioid, clear cell, mucinous] or type II (HGSC, undifferentiated) OC patients (Table 1; Supplementary Table S1, S3; Fig. 1) ^40^.

Seventeen patients (13.8%) carried a GPV in HBOP cancer predisposition genes, including only a single carrier of GPV in *BRCA1* and *BRCA2*, respectively (2/123; 1.6%), compared to 30.2% GPV in population-matched OC patients published previously (Table 2)^6^. We found no GPV in established genes associated with the risk of non-epithelial tumors of ovary (*STK11, SMARCA4, DICER1*).

**Table 2:** GPV in established hereditary breast/ovarian/pancreatic (HBOP) cancer predisposition genes identified in early-onset OC patients and comparison of GPV frequency in unselected OC patients.

Gene	123 early-onset OC N (%)	1320 unselected OC^6^* N (%)	*p* value
*ATM*	2 (1.6)	6 (0.5)	0.14
*BARD1*	2 (1.6)	3 (0.2)	0.06
***BRCA1***	1 (0.8)	229 (17.4)	**8.5 × 10**^**–9**^
***BRCA2***	1 (0.8)	94 (7.1)	**0.003**
BRIP1**	1 (0.8)	10 (0.8)	1
*CHEK2*	6 (4.9)	12 (0.9)***	**0.002**
***MLH1***	0	4 (0.3)	1
***MSH2***	1 (0.8)	3 (0.2)	0.3
*MSH6*	0	3 (0.2)	1
*PALB2*	0	8 (0.6)	1
*PMS2*	1 (0.8)	NA	NA
***RAD51C***	2 (1.6)	13 (1)	0.37
***RAD51D***	0	13 (1)	0.62
TP53**	1 (0.8)	1 (0.1)	0.16
***All***	***17 (13.8)***	***399 (30.2)***	***6.6* × *10***^***–5***^

*multiple GPV carriers (N = 13) were excluded from the analysis; **one concomitant carrier of GPV in BRIP1 and TP53; ***additionally, one initially unrevealed deep intronic GPV in CHEK2 was identified.

GPV, Germline Pathogenic Variants; NA, not available; OC, Ovarian Cancer.

Established high-penetrance OC predisposition genes and significant *p* values are in bold.

**Figure 2:**
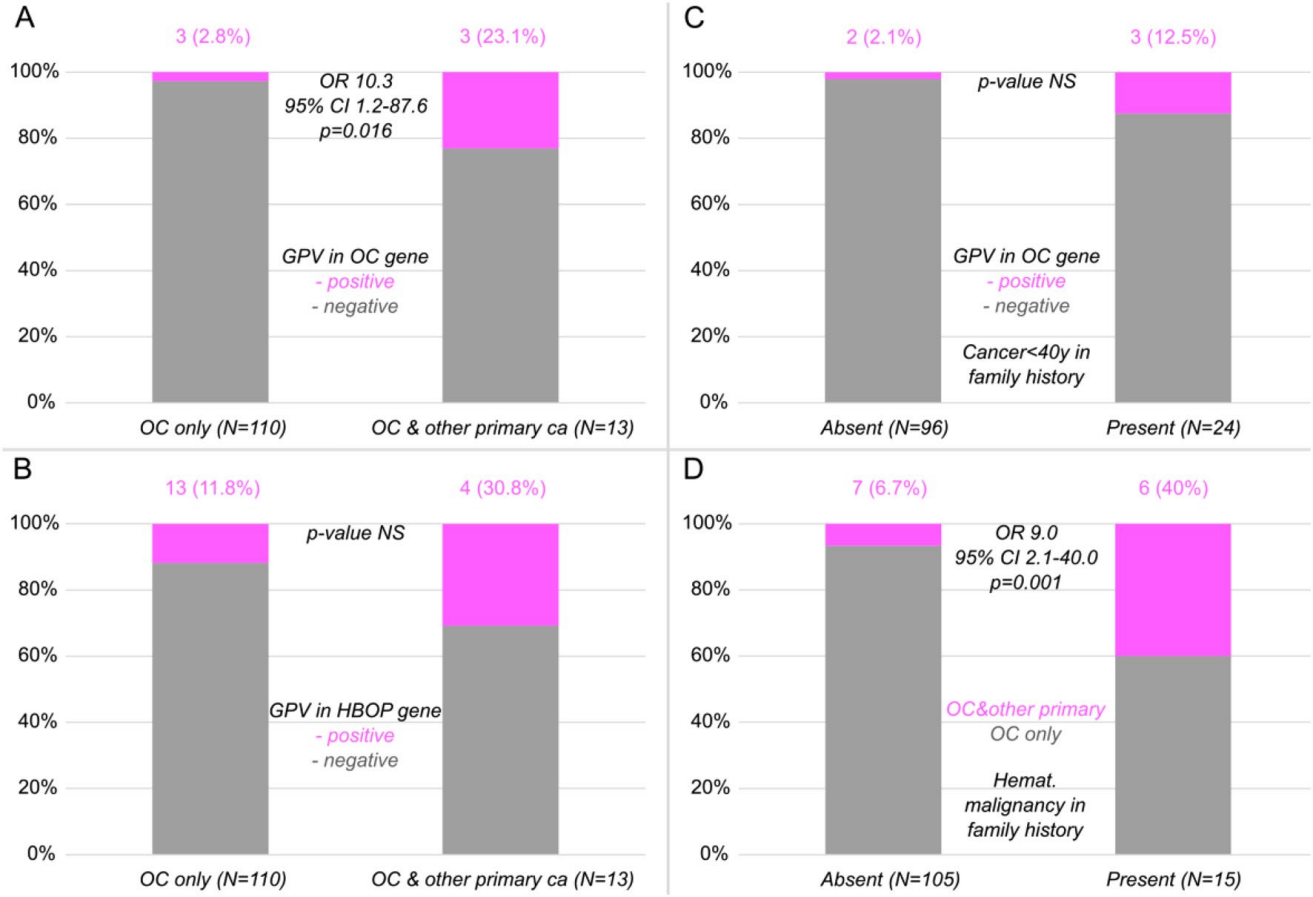
Association of germline pathogenic variant (GPV) in ovarian cancer (OC; **A**) and hereditary breast/ovarian/pancreatic (HBOP; **B**) cancer predisposition genes with multiple primary tumors in early-onset OC patients, and with family cancer history (**C**), and association of positive personal cancer history beyond OC with hematological malignancies in family cancer history (**D**). CI, Confidence Interval; NS, not significant; OR, Odds Ratio.

The carriership of GPV in established high-penetrance OC predisposition genes (Table 2; in bold) was significantly associated with development of multiple primary tumors (*p* = 0.016; Fig. 2A), whereas GPV in established HBOP cancer predisposition genes (Table 2; Fig. 2B) was not, presumably due to the prevalence of moderate penetrance GPV carriers. GPV in established high-penetrance OC predisposition genes (Table 2; in bold) associated neither with early-onset (< 40 years) malignancy in family cancer history(*p* = 0.054; Fig. 2C), nor with overall positive family cancer history, nor with hematological malignancies in family history, which was observed in the initial study of Stratton et al.^7^. On the other hand, multiple primary tumors in early-onset OC patients significantly associated with family history of hematological malignancies (*p* = 0.001; Fig. 2D). It is noteworthy that among early-onset OC patients with known histology, double primaries developed more likely in early-onset OC patients diagnosed with non-epithelial OC (5/15; 33.3%) than with invasive epithelial OC (4/61; 6.6%; *p* = 0.012; Supplementary Table S1).

WES gene-based burden analysis revealed *CHEK2* and *LY75-CD302* (6 carriers each) as the most frequently altered genes in early-onset OC patients (*p* = 1.2 × 10^–4^ and 8.3 × 10^–4^, respectively). Altogether, GPV in only five genes (*CHEK2, LY75-CD302, ATP7B, BCHE, MFNG)* were enriched in early-onset OC patients compared to unselected controls (Supplementary Table S7); however, the significance did not fall below the exome-wide Bonferroni corrected *p*-value threshold.

Of these genes, *CHEK2* was the only one associated with cancer risk. Interestingly, alongside to known founder c.1100delC and exons 9–10 deletion GPV, RNA analysis revealed two intronic GPV, c.1009-118_1009-87delinsC and c.1461 + 2301G > T, leading to the aberrant splicing (Supplementary Table S3). The recurrent c.1009-118_1009-87delinsC variant has been recently reported^41^. The deep intronic c.1461 + 2301G > T variant has been previously assessed as variant of uncertain significance (VUS) in ClinVar due to its predicted in-frame insertion of 30 amino acids. However, our RNA analysis revealed a new acceptor splice site leading to an inclusion of 89 nucleotides (r.1461_1462ins1461 + 2211_1461 + 2299) and predicted premature termination of translation (p.Asp488SerfsTer34; Supplementary Fig. S1). Interestingly, the age at OC diagnosis significantly differed between *CHEK2* carriers and non-carriers of GPV in HBOP cancer predisposition genes^6^ (median age 33.9; 18–66 years, and 58; 15–92 years, respectively; *p* = 0.003). The *CHEK2* status did not associate with a particular OC histology; most (4/6) *CHEK2* GPV carriers had a positive family cancer history (Supplementary Table S1, S4; Supplementary Fig. S2).

The second most frequently altered gene was *LY75-CD302* involved in immune response. GPV in *LY75-CD302* gene were unique except for c.4503del identified in two OC patients (Supplementary Table S3). Interestingly, four out of six *LY75-CD302* GPV affected exon 31 that codes for C-type lectin/C-type lectin-like domain important for antigen binding prior to endocytosis and antigen presentation^42,43^. All the carriers of *LY75-CD302* GPV had a positive family cancer history, and none of them developed multiple primary tumors; *LY75-CD302* GPV did not associate with any specific OC histology (Supplementary Table S1, S3).

All GPV were present in heterozygous state, with the exception of two patients carrying two GPV in the same gene (*MUSK* and *MAATS1*) suggesting possible recessive inheritance; however, their phase was unknown.

To determine whether a pre-defined set of genes belonging to certain pathway or disease are over-represented in our OC patients, we performed overrepresentation analysis of the genes from final list of GPV (Supplementary Table S3) using functional databases. A combined analysis could be empowered if multiple genes of a predefined set were associated but the effect size is too small to detect individually. The overrepresentation analysis showed highest enrichment for gene set #114,480: Breast cancer (disease in OMIM; *p* = 4.5 × 10^–10^; FDR 3 × 10^–9^; Supplementary Fig. S3) followed by gene set #HP:0,030,406 Primary peritoneal carcinoma (phenotype in Human Phenotype Ontology; *p* = 6.7 × 10^–6^; FDR 0.011).

### HLA genotypization

We analyzed HLA of class I (HLA-A, B, C) and class II (HLA-DPA1, DPB1, DQA1, DQB1, and DRB1) in 123 patients (except for HLA-DRB1 not genotyped in two patients due to the insufficient coverage of sequencing data in the HLA-DRB1 region). To increase robustness of the analysis, HLA alleles frequencies were compared to two sets of controls (HLA- and super-controls). Two HLA class I (HLA-A*36:01 and HLA-B*53:01) and three HLA class II (HLA-DRB1*11:01, HLA-DQA1*01:03 and HLA-DQA1*03:03) alleles were significantly enriched in patients compared to HLA- and super-controls (Supplementary Table S8).

**Figure 3:**
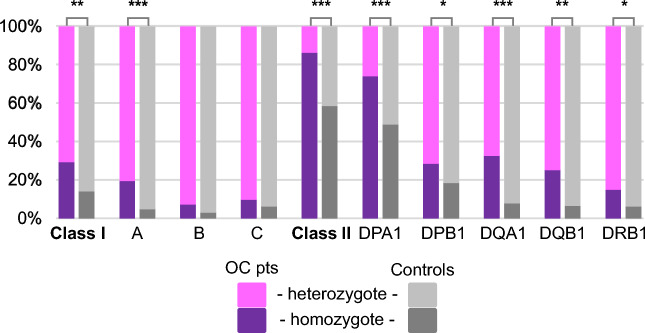
Frequency of Human Leukocyte Antigen (HLA) loci homozygotes in early-onset ovarian cancer (OC) patients compared to female super-controls. *p < 0.05; **p < 0.001;***p < *10*^*–5*^*.*

We assessed the ratio of homozygotes in patients compared to super-controls in each locus. Patients were significantly more frequently carriers of at least one homozygous HLA allele (*p* = 3 × 10^–7^). The carriers’ frequency of multiple homozygous alleles was also significantly increased (Supplementary Table S9). The rates of homozygotes in HLA class I and II were both statistically significantly higher compared to super-controls (Fig. 3).

### PRS analysis

**Figure 4:**
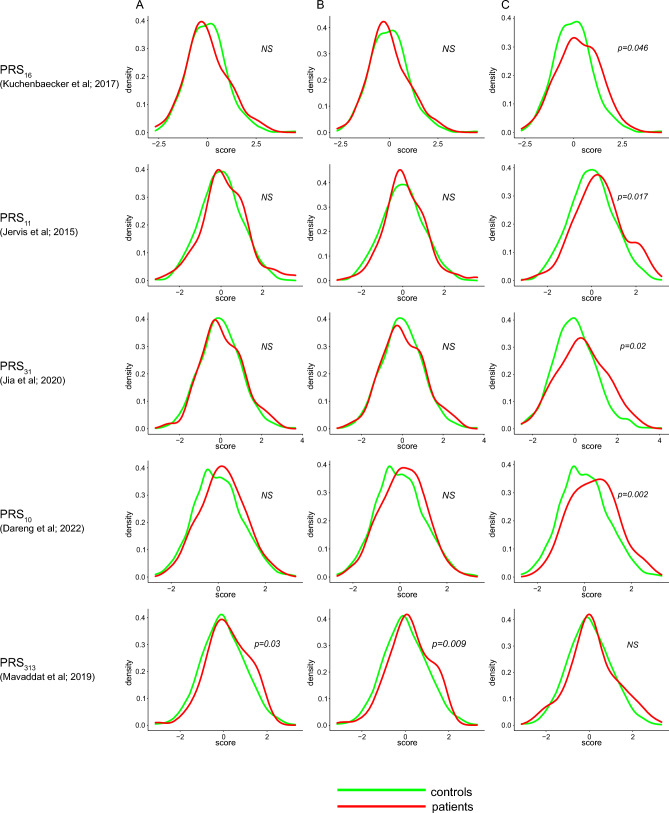
Comparison of polygenic risk scores (PRS) distribution between subgroups of ovarian cancer (OC) patients and PRS controls. Only SNP sets significantly stratifying at least one subgroup of OC patients from PRS controls are shown; (**A**) early-onset OC patients, (**B**) pooled early-onset and histology/stage-matched OC patients, (**C**) late-onset high-grade serous OC (HGSC) patients. Details are provided in Supplementary Table S5. *NS, not significant.*

In contrast to BC, a single SNP set has not yet been widely adopted for the analysis of PRS in OC, although many have been published and, therefore, we performed PRS analysis using 10 different (rather) non-overlapping SNP sets associated with OC ^12–21^. However, PRS of early-onset OC differed significantly neither from PRS controls, nor from histology/stage-matched OC patients in any individual OC SNP set analyzed (Supplementary Table S5; Fig. 4). Similarly, we did not observe any difference between PRS of histology/stage-matched OC patients and PRS controls.

The SNP sets selection is usually based on studies of patients regardless of OC histology. Since HGSC is the most common and PRS did not differ between non-HGSC OC patients and PRS controls, we analyzed PRS in late-onset HGSC patients who tested negative for GPV in OC predisposition genes to evaluate the SNP sets for PRS calculation^44^ (Supplementary Table S4) to evaluate the performance of the individual SNP sets for PRS calculation in HGSC patients compared to PRS controls. Contrary to early-onset predominantly non-HGSC OC, PRS of HGSC patients based on 4 individual SNP sets differed significantly from PRS controls (Supplementary Table S5). Additionally, Phelan et al*.* established OC histology-specific OR for each included SNP (Supplementary Table S2). However, when we used LGSC or mucinous-specific OR for PRS calculation in 64 and 28 OC patients (pooled early- and late-onset patients), the PRS did not differ from that of PRS controls.

Furthermore, we calculated PRS using 313 SNP with OR established for BC. Surprisingly, the difference in PRS between both early-onset and pooled early-onset and histology/stage-matched OC patients (thus, predominantly non-HGSC OC), and PRS controls was significant (*p* = 0.03 and 0.009, respectively) whereas non-significant in HGSC (Supplementary Table S5; Fig. 4). The PRS_313_ significantly stratified early-onset OC from controls even after excluding six early-onset OC patients with double primary BC (*p* = 0.02). The representation of early-onset OC patients in each decile according to PRS_313_ was quite even. Interestingly, six early-onset OC patients were also diagnosed with BC and PRS_313_ categorized them into 1st, 3rd, 5th,7th, 9th, 9th decile, respectively; the patient carrying a *BRCA2* GPV was categorized in the 1st decile.

### Survival analysis

**Figure 5:**
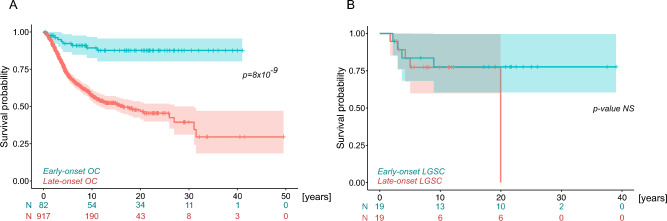
Survival analysis of ovarian cancer (OC) patients. Kaplan–Meier survival curves of (**A**) early-onset and late-onset OC patients; (**B**) Low-grade serous (LGSC) early- and late-onset OC. *NS, not significant.*

Finally, we performed a survival analysis to pursuit a difference in early- and late-onset OC patients. Regardless of their GPV carriership, the survival analysis of 82 early-onset and 917 late-onset OC patients with available vital status showed significantly improved survival of early-onset OC patients (*p* = 8 × 10^–9^; Fig. 5A). The survival differed significantly according to germline *gBRCA1/2* status (*p* = 0.002). However, we can only comment in principle on late-onset OC patients as g*BRCA1/2* GPV were rare among early-onset OC (216/917; 23.6% vs. 2/82; 2.4%). Survival analysis of 80 *gBRCA1/2*-negative early-onset (median age 25.1 years) and 79 histology/stage-matched *gBRCA1/2*-negative OC (median age 61.7 years) was also significant although the survival advantage was less pronounced (*p* = 0.02). Survival analysis for individual histological subtypes was not significant (LGSC; Fig. 5B or cannot be performed due to insufficient number of events in at least one of the patients` groups.

## Discussion

The early-onset OC (diagnosed at < 30 years), constitutes a distinct subgroup that differs markedly from late-onset OC in various aspects, including germline cancer predisposition. We undertake the most extensive germline analysis of early-onset OC patients so far, employing comprehensive approaches encompassing DNA WES complemented by RNA WES and PRS analysis.

Mutation profiles in our early-onset OC patients were dissimilar to unselected predominantly HGSC patients that we analyzed previously^6^, with *BRCA1/BRCA2* GPV identified in only two (1.6%) early-onset OC patients. Our observation aligns with findings from the limited studies that have investigated early-onset OC patients, which consistently report either the absence or an unusually low frequency of GPV in established OC predisposition genes, as reviewed in^11^. It is noteworthy that GPV in established high-penetrance OC predisposition genes in our study significantly associated with multiple primary malignancies in early-onset OC patients who tended to develop invasive OC, and were more likely diagnosed with non-epithelial OC. The increased risk of double primary tumors was observed in a large study by Casper et al*.* who described the inverse correlation between age at OC diagnosis and double primary cancer risk^45^. Moreover, the risk of double primary tumors was not increased in patients with BTO in a SEER population-based study^46^. Notably, early-onset OC patients with double primary tumors in our study had a significantly higher likelihood of hematological malignancies in their family history, consistent with Stratton et al.'s study, which increased risk of non-Hodgkin lymphoma and malignant myeloma in first degree relatives of early-onset OC patients with invasive disease^7^.

Most GPV were identified uniquely in our early-onset OC cohort and were enriched in only five genes compared to unselected controls, with the *CHEK2* gene coding for checkpoint kinase 2 ranking first. Interestingly, *CHEK2* GPV identified in 6/123 (4.9%) early-onset OC patients were significantly associated with earlier age at diagnosis compared to previously analyzed OC patients negative for GPV in HBOP cancer predisposition genes^6^. It is noteworthy that one *CHEK2* deep intronic variant was identified only by RNA NGS which underlines the importance of RNA analysis^47^. Furthermore, GPV in *CHEK2* have been identified in early-onset OC patients by other studies^6,10,48,49^, the most prevalently by Carter et al.^10^ who identified *CHEK2* GPV in 5/147 (3.4%) early-onset OC patients. Although *CHEK2* have not been acknowledged as the OC predisposition gene, some studies pointed to an association of *CHEK2* with the OC risk^6,50,51^. GPV in *CHEK2* are associated with moderate BC risk^52^. Interestingly, our overrepresentation analysis identified a BC gene set enrichment in carriers of GPV in our early-onset OC patients. In addition, PRS_313_ developed for BC risk stratification significantly differed in early-onset OC patients compared to PRS controls (see below). Moreover, 5/13 early-onset OC patients with a second primary tumor were diagnosed with BC. Consequently, it is plausible to speculate that there may be a shared germline predisposition factor(s) or common underlying mechanisms to both BC and early-onset OC, particularly non-HGSC OC.

Besides *CHEK2*, *LY75-CD302* was the second most GPV-enriched gene revealed by the gene burden analysis. This gene consists of *LY75* and *CD302* that are alternatively transcribed in a readthrough way leading to translation of fusion proteins with high similarity, acting as receptors involved in endocytosis-mediated immune responses including HLA class I-mediated antigen presentation ^42,53^. In addition, *LY75* was shown to modulate cellular phenotype of epithelial OC cells and their metastatic potential through mediation of mesenchymal-epithelial transition^54,55^. Although the significance of GPV in *LY75-CD302* (and other immunity-related genes with identified private GPV) for early-onset OC risk remains unknown, these findings suggest a promising avenue for research in early-onset OC development, particularly considering also the results of the HLA analysis.

HLA molecules are coded by multiple highly polymorphic loci and are crucial for immune reaction activation and progression including anti-tumor immunity^56^. Previously, specific HLA alleles have been described to predispose to certain cancer types, including OC, as observed by Kubler et al*.* who identified a significantly higher frequency of carriers of HLA class II haplotypes HLA-DQA1*05:01-DQB1*02:01-DRB1*03:01 and HLA-DQA1*01:01-DQB1*05:01-DRB1*10:01) in OC patients from Germany^22^. In addition, HLA-DRB1*03:01 (homozygous or heterozygous) and HLA-DQB1*02:01 (only homozygous) were individually enriched in their patients compared to controls. However, we did not observe significant enrichment of either these haplotypes or genotypes in our cohort; however, we identified another HLA-DRB1 (*11:01) allele enriched in our early-onset OC patients. This allele was associated with BC in Italian cohort of patients previously^57^. The high abundance of HLA-DRB1*11:01 carriers among our early-onset OC patients might indicate a genetic link between BC, immune system, and early-onset OC. Additionaly, we identified significantly associated HLA-DQA1*01:03 risk allele and HLA-DQA1*03:03 protective allele. However, the cancer risk associations of HLA-DQA1 are contradictory and not very well understood yet^22,58,59^.

In addition to the specific disease-related risk HLA alleles, the heterogeneity of inherited HLA alleles also appears to be important in anti-tumor immunity^56^. Having analyzed the HLA zygosity, the early-onset OC patients were significantly more frequently homozygotes compared to super-controls. Interestingly, homozygotes in HLA class II were more abundant than homozygotes in HLA class I loci when compared to super-controls. HLA homozygosity was previously associated with increased risk of lung and head and neck cancer, and lymphomas, thus, particularly with tumors with high mutational burden or infectious etiology^60^.

Polygenic inheritance, arising from the cumulative effect of numerous genetic low-risk variants, might elucidate a portion of the missing heritability in the predisposition to early-onset OC^61^. However, PRS analysis has currently its limitations, given the lack of consensus on a specific SNP set and uncertain clinical efficacy in OC risk stratification^13,62^. We conducted PRS analysis using 10 different SNP sets^12–21^, but none demonstrated the ability to distinguish early-onset from histology/stage-matched OC patients, or from PRS controls. Conversely, PRS based on four SNP sets^13,15,16,21^ were able to discriminate between HGSC patients and PRS controls. This implies that these four SNP sets are specifically associated with the risk of HGSC, the most prevalent OC type in GWAS focused on identifying OC risk loci. Interestingly, PRS_313_, designed specifically for BC^38^, significantly differed in early-onset, predominantly non-HGSC OC patients but not in HGSC. This suggests a potential pleiotropic effect, indicating a common mechanism underlying the development of multiple phenotypes associated with some common variant susceptibility loci. However, evidence supporting a biological function has only been identified for certain loci, leaving a significant portion of biology still unclear.

Considering polygenic inheritance from an alternative perspective, we observed significant variability in GPV burden among different OC types and super-controls. Higher GPV burden was identified in patients diagnosed with invasive epithelial OC and, remarkably, GPV burden tended to increase with increasing somatic genomic instability characteristic for each histological OC types^40,63^. Interestingly, Qing et al*.* noticed strong negative correlation between GPV burden and age, suggesting a greater contribution of GPV to the transformation process in early-onset OC patients compared to their late-onset counterparts, where somatic mutations were hypothesized to play a more predominant role^64^. This observation supports our hypothesis that, the cumulative GPV burden may potentially elevate the cancer risk rather than GPV in certain genes, particularly when concomitant with reduced HLA diversity, influencing the efficiency of neoantigen recognition.

Regarding to the association of clinicopathological and genetic factors with the survival, we observed an improved survival of early-onset OC patients compared to previously analyzed late-onset OC patients^6^. In addition, our findings revealed survival advantage in early-onset OC patients compared to histology/stage-matched OC patients lacking *gBRCA1/2* GPV. This suggests that age is an independent positive prognostic factor. A positive correlation between survival and age as well as improved survival among non-HGSC epithelial OC patients has been described in prior research^65,66^. Nevertheless, certain investigations have delineated a less favorable prognosis and lower 5-year survival in LGSC OC in early-onset OC^67^. However, we did not observe any difference in survival of LGSC early- and late-onset OC patients.

While our study stands out as the most intricate and the third-largest investigation focused on early-onset OC patients diagnosed before the age of 30, still a noteworthy limitation lies in the restricted number of patients. The low number of patients hinders our ability to pinpoint potential private causal alleles effectively. These limitations underscore the need for comprehensive data to better understand the complex landscape of early-onset OC and its associated risk factors.

In conclusion, our comprehensive germline analysis of early-onset OC patients revealed two divergent trajectories of potential germline susceptibility. Overrepresentation analysis highlighted an association to BC, supported by the enrichment of GPV in *CHEK2* and the presumably BC-specific PRS_313_, which successfully stratified early-onset OC from PRS controls. The second avenue pointed towards the impaired immune response, indicated by GPV in the *LY75-CD302* gene, coupled with diminished HLA diversity. Furthermore, we found a significantly higher GPV burden in early-onset OC patients compared to super-controls.

In summary, the genetic predisposition to early-onset of OC appears to be a very heterogeneous and complex process beyond the conventional Mendelian monogenic understanding of hereditary cancer predisposition with a modifying role of the immune system. Based on our results, we speculate that rather a cumulative GPV burden than GPV in specific genes may increase early-onset OC risk, especially when it is concomitant with reduced HLA diversity, which affects the efficiency of neoantigen recognition. However, it cannot be excluded that the occurrence of early-onset OC is a stochastic event influenced by random variables.

### Supplementary Information

Supplementary Tables.

Supplementary Figures.

Supplementary Information.

## Data Availability

The authors confirm that the data supporting the findings of this study are available within the article in its supplementary materials. The raw data cannot be publicly shared due to the GDPR policy.

## Abbreviations

BC: Breast cancer
BTO: Borderline tumors of ovary
CI: Confidence interval
CNV: Copy number variations
FDR: False discovery rate
*gBRCA1/2*: Germline *BRCA1/2*
GPV: Germline pathogenic variants
HBOP: Hereditary breast, ovarian and pancreatic
HGSC: High-grade serous carcinoma
HLA: Human leukocyte antigen
LGSC: Low-Grade serous carcinoma
NA: Not available
neg.: Negative
NGS: Next generation sequencing
NS: Not significant
NOS: Not otherwise specified
OC: Ovarian cancer
OR: Odds ratio
PRS: Polygenic risk score
SNP: Single nucleotide polymorphism
SNV: Single nucleotide variant
VUS: Variant of uncertain significance
WES: Whole exome sequencing
