# Supplementary material for: A comprehensive analysis of germline predisposition to early-onset ovarian cancer
Source: Sci Rep. 2024 Jul 13;14:16183. doi: 10.1038/s41598-024-66324-2 (PMC11246516; doi:10.1038/s41598-024-66324-2)
Supplement: Supplementary file 2 — Supplementary Figures. [file 41598_2024_66324_MOESM2_ESM.pptx]

## Slide 1
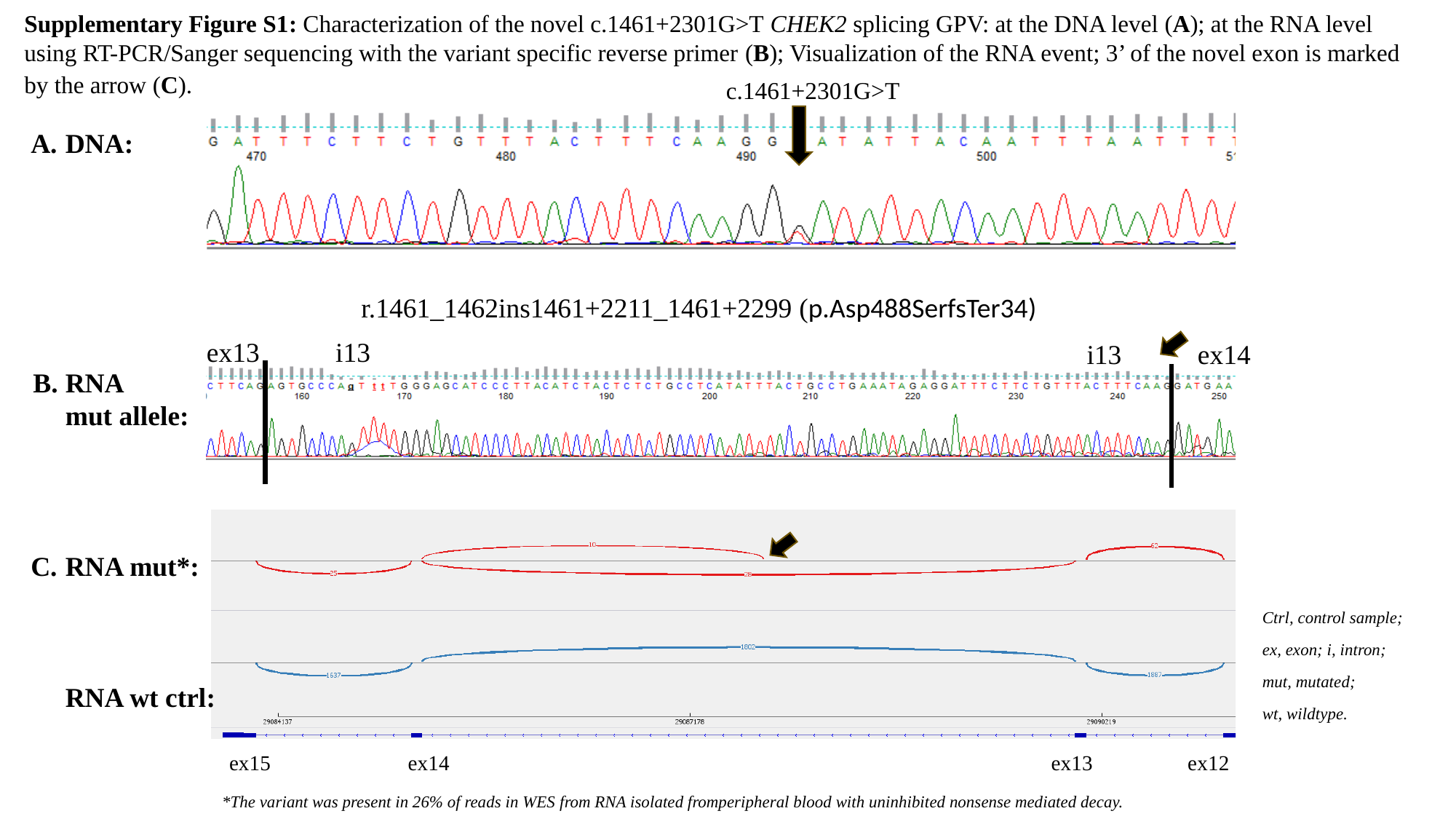

Supplementary Figure S1: Characterization of the novel c.1461+2301G>T CHEK2 splicing GPV: at the DNA level (A); at the RNA level using RT-PCR/Sanger sequencing with the variant specific reverse primer (B); Visualization of the RNA event; 3’ of the novel exon is marked by the arrow (C).
c.1461+2301G>T
A.
DNA:
r.1461_1462ins1461+2211_1461+2299 (p.Asp488SerfsTer34)
ex13 i13
i13 ex14
B.
RNA
mut allele:
C.
RNA mut*:
RNA wt ctrl:
Ctrl, control sample;
ex, exon; i, intron;
mut, mutated;
wt, wildtype.
ex15 ex14 ex13 ex12
*The variant was present in 26% of reads in WES from RNA isolated fromperipheral blood with uninhibited nonsense mediated decay.

## Slide 2
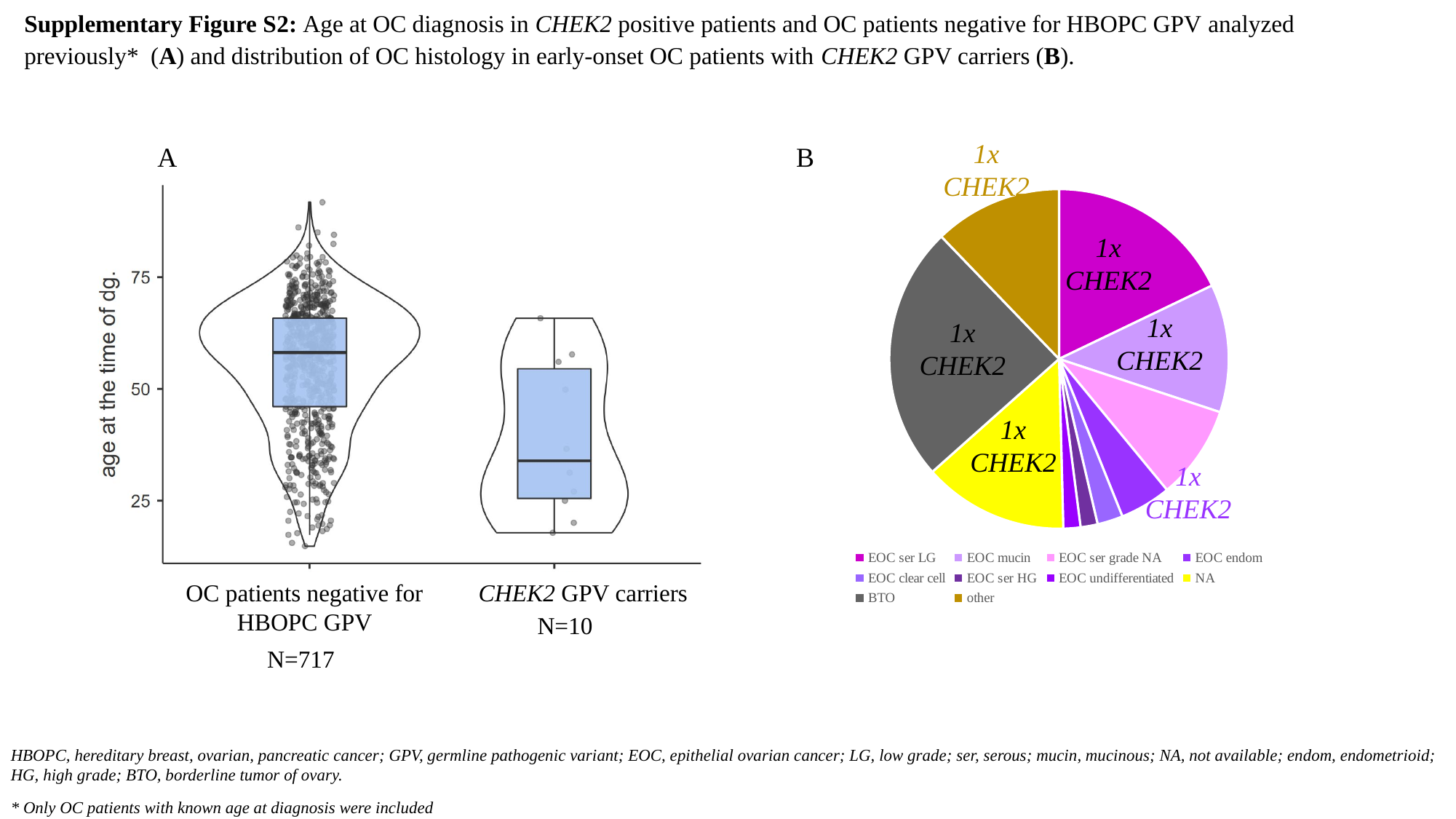

Supplementary Figure S2: Age at OC diagnosis in CHEK2 positive patients and OC patients negative for HBOPC GPV analyzed previously* (A) and distribution of OC histology in early-onset OC patients with CHEK2 GPV carriers (B).
1x CHEK2
A
B
### Chart
| Category | |
|---|---|
| EOC ser LG | 22.0 |
| EOC mucin | 15.0 |
| EOC ser grade NA | 11.0 |
| EOC endom | 6.0 |
| EOC clear cell | 3.0 |
| EOC ser HG | 2.0 |
| EOC undifferentiated | 2.0 |
| NA | 17.0 |
| BTO | 30.0 |
| other | 15.0 |1x CHEK2
1x CHEK2
1x CHEK2
1x CHEK2
1x CHEK2
CHEK2
OC patients negative for HBOPC GPV
CHEK2 GPV carriers
N=10
N=717
HBOPC, hereditary breast, ovarian, pancreatic cancer; GPV, germline pathogenic variant; EOC, epithelial ovarian cancer; LG, low grade; ser, serous; mucin, mucinous; NA, not available; endom, endometrioid; HG, high grade; BTO, borderline tumor of ovary.
* Only OC patients with known age at diagnosis were included

## Slide 3
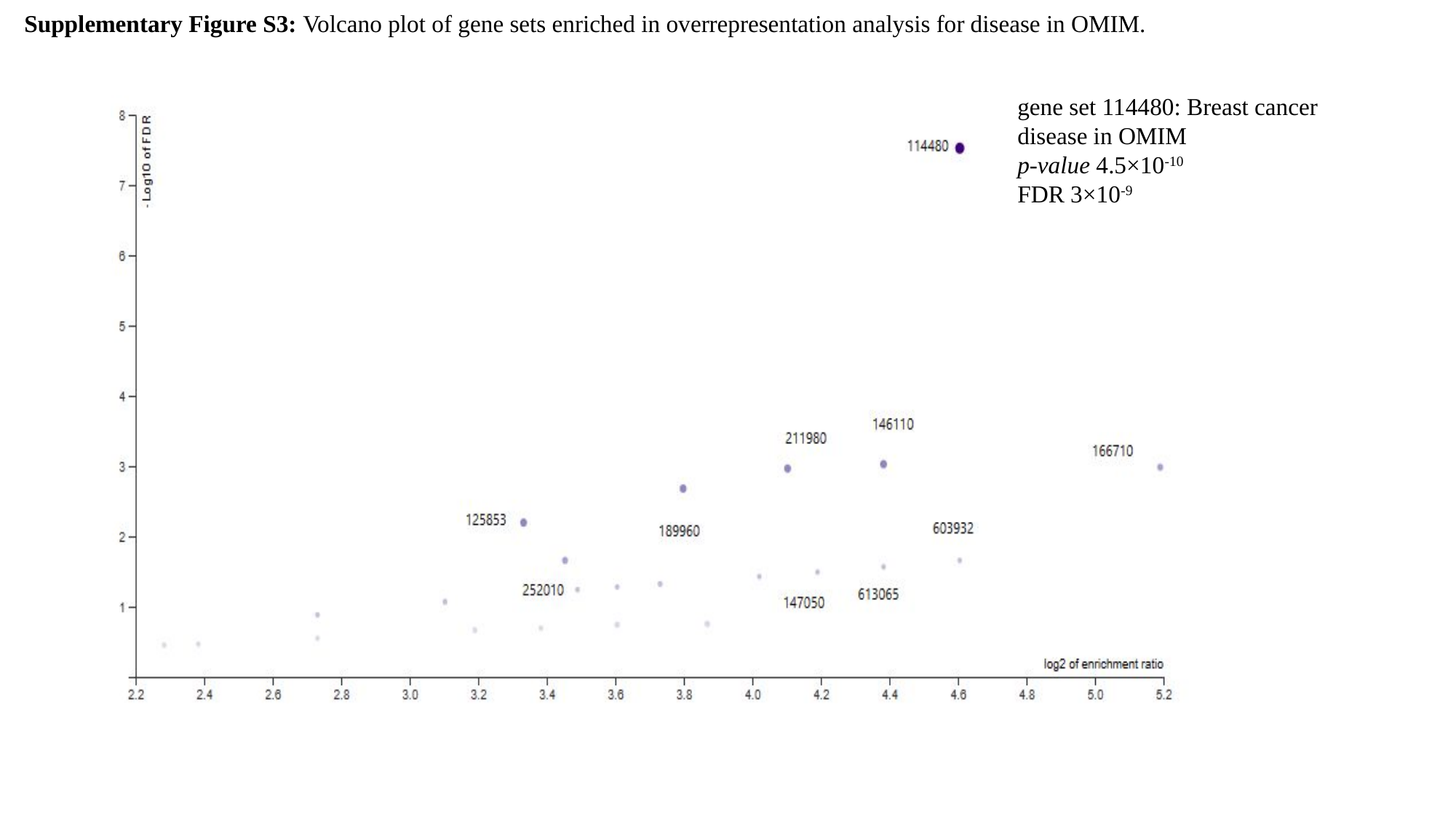

Supplementary Figure S3: Volcano plot of gene sets enriched in overrepresentation analysis for disease in OMIM.
gene set 114480: Breast cancer
disease in OMIM
p-value 4.5×10^-10^
FDR 3×10^-9^

## Slide 4
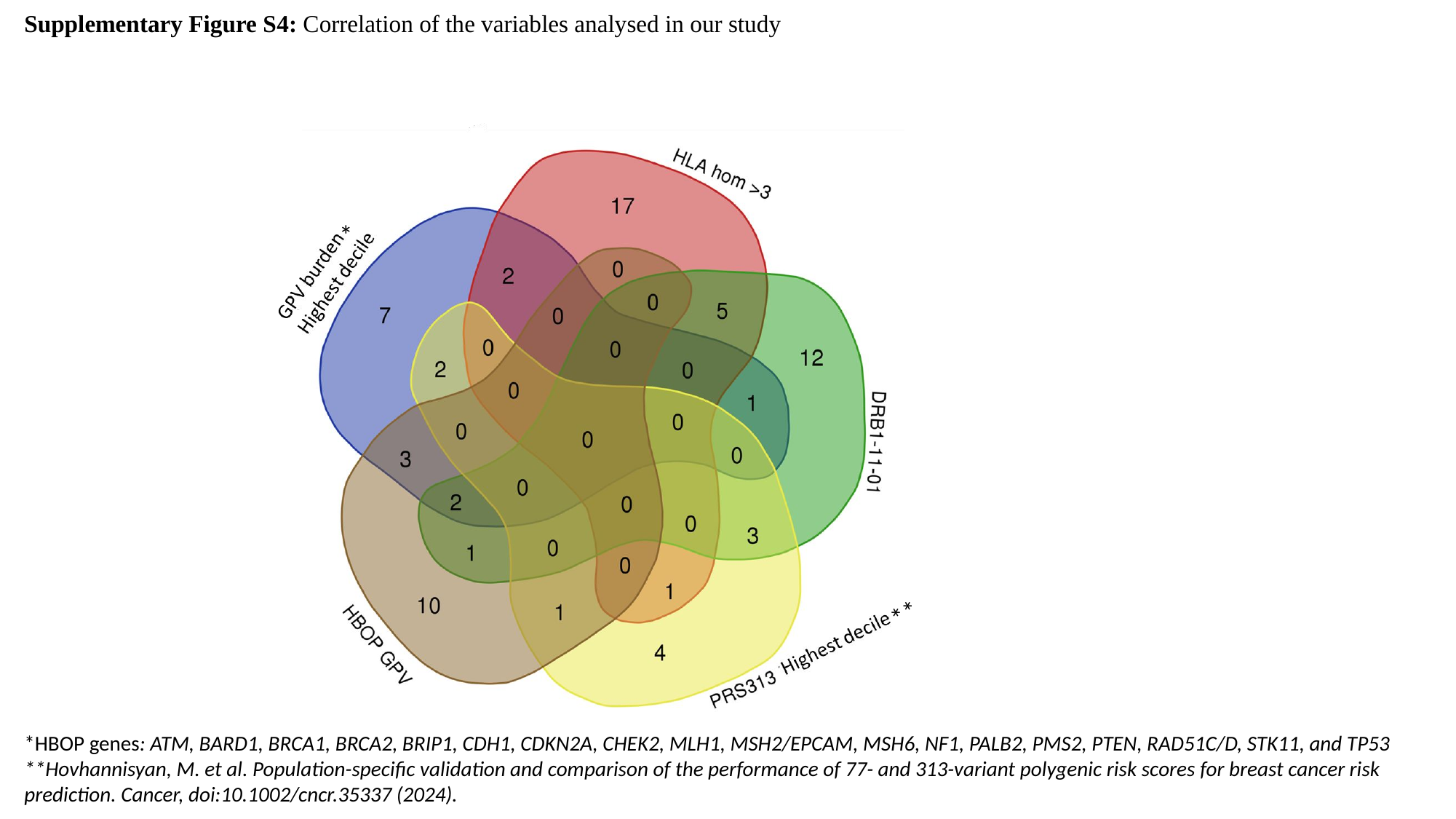

Supplementary Figure S4: Correlation of the variables analysed in our study
*
**
*HBOP genes: ATM, BARD1, BRCA1, BRCA2, BRIP1, CDH1, CDKN2A, CHEK2, MLH1, MSH2/EPCAM, MSH6, NF1, PALB2, PMS2, PTEN, RAD51C/D, STK11, and TP53
**Hovhannisyan, M. et al. Population-specific validation and comparison of the performance of 77- and 313-variant polygenic risk scores for breast cancer risk prediction. Cancer, doi:10.1002/cncr.35337 (2024).
