# Supplementary material for: A comprehensive analysis of germline predisposition to early-onset ovarian cancer
Source: Sci Rep. 2024 Jul 13;14:16183. doi: 10.1038/s41598-024-66324-2 (PMC11246516; doi:10.1038/s41598-024-66324-2)
Supplement: Supplementary file 3 — Supplementary Information. [file 41598_2024_66324_MOESM3_ESM.docx]

**Early-onset ovarian cancer: a comprehensive analysis**

Klara Horackova, Petra Zemankova, Petr Nehasil, Michal Vocka, Milena Hovhannisyan, Katerina Matejkova, Marketa Janatova, Marta Cerna, Petra Kleiblova, Sandra Jelinkova, Barbora Stastna, Pavel Just, Tatana Dolezalova, Barbora Nemcova, Marketa Urbanova, Monika Koudova, Jana Hazova, Eva Machackova, Lenka Foretova, Viktor Stranecky, Michal Zikan, Zdenek Kleibl, Jana Soukupova

# Supplementary Methods

# Patients and controls

Positive family cancer history included several categories (Supplementary Table S1): i) HBC included breast cancer diagnosed before age 50 in a first or second-degree relative, or ≥2 breast cancer cases in family history; ii) HOC was defined as OC at any age in first or second-degree relatives and without breast cancer in family history; iii) HBOC encompassed both the breast and ovarian cancer in family history; iv) the "other" category included any other types of cancers in the family history beyond breast and ovarian cancer.

# Library Preparation

Sequencing libraries were prepared as described previously [1-3] with minor modification. Briefly, fragmented gDNA (by ultrasound or enzymatically; mean fragment length 200 bp) was used for DNA library preparation using either KAPA HyperPrep Kit, KAPA HyperPlus Kit or KAPA EvoPlus Kit (all Roche). RNA libraries were prepared using KAPA RNA HyperPrep Kit (Roche). Plexes of up-to 36 barcoded samples (2 µg in total) were hybridized with KAPA HyperExome panel (capture target 43Mb; Roche). The final libraries were sequenced on the NovaSeq 6000 system using NovaSeq SP, S1, or S2 Reagent Kit v1.5, 200 cycles (Illumina). The minimal mean coverage was 30× for DNA and 200× for RNA in exon 11 of the *BRCA1* gene that was selected as a coverage quality marker.

In addition, plexes of up-to 96 barcoded samples were hybridized with custom HyperChoice panel (Roche) targeting 843 single nucleotide variants (SNV) [4] including 65 SNV previously reported to associate with ovarian cancer (OC) polygenic risk score (PRS; Supplementary Table S2). The final libraries were sequenced on the NextSeq 500 system using NextSeq 500/550 Mid Output Kit v2.5, 150 cycles (Illumina). The minimal mean coverage was 20×.

Bioinformatical analysis was performed as described previously [1-3] with minor modifications – variant filtration was performed with minimal fraction of alternative allele equal to 25%; GATK HaplotypeCaller 3.8, and SnpEff 4.3 tools were used for variant calling and for annotation and variant effect prediction, respectively.

# Variant filtration and prioritization for the gene burden analysis

## DNA substitutions/short-medium length indels

Called variants from DNA whole exome sequencing (WES) of patients were filtered out according to the following criteria:

1. sequencing quality <150;
2. located in repetitive and low-complexity regions (based on RepeatMasker [5]);
3. located in non-coding regions (downstream, intergenic, intron, upstream gene variants) or leading to in-frame insertion/deletion;
4. present in „super-controls“ >0×;
5. present in population databases (gnomAD, 1000 Genomes Project, NHLBI GO ESP, ExAC [6-8]) >0,1 %;
6. classified in ClinVar database [9] as benign or likely benign;
7. located in last exons;
8. inspected in Integrated Genome Viewer (IGV) and considered as sequencing error;
9. unless classified in ClinVar database [9] as pathogenic/likely pathogenic.

Subsequently, prioritized variants were classified pathogenic/likely pathogenic (Supplementary Table S3) when fulfilling at least one of the following criteria:

1. variants leading to premature termination, start-loss or frameshift;
2. splicing variants located 1-2 bp from the intron/exon boundary;
3. splicing variants predicted as causing aberrant splicing by Splicing Prediction Pipeline (SPiP) [10] <85% and SpliceAI in any parameter <10 [11];
4. splicing variants with lower prediction markers inspected in RNA leading to at least 10% of aberrant splicing;
5. variants classified in ClinVar database [9] as pathogenic/likely pathogenic (at least two submitters).

Prioritization resulted in identification of 1438 pathogenic/likely pathogenic variants that include 834 variants causing frameshift or premature termination, 93 missense variants previously reported in ClinVar as pathogenic/likely pathogenic, 34 start-loss variants, 182 splicing variants localized within the canonical splice site (± 1-2 bp from the intron/exon boundary), and 295 variants predicted by SPiP, and SpliceAI as deleterious.

## Copy number variation (CNV) analysis

CNV analysis was coverage-based, performed using modified CNV kit as described previously [1]. We prioritized events with the normalized CNV score <-0.8 or >0.8 for deletion and duplication, respectively, that occurred in ≤2 patients and were absent in super-controls; patients with >8000 events were excluded. We obtained 2316 events. To avoid high false positivity, only events affecting ≥2 exons were considered. Whole gene duplication of duplication affecting cDNA boundaries were considered VUS.

CNV prioritization resulted in identification of 40 patients with suspicious deletions and 6 patients with suspicious duplications of two or more exons in the same gene. The suspicious CNV were manually inspected in IGV having, finally, selected 15 CNV (14 deletions and 1 duplication) in 17 patients (two patients harbored the same large deletion in *CHEK2* and *ACAD11).* In addition, 5 CNV (all deletions) in 5 patients were identified among RNA events (Supplementary Table S3).

## RNA events

Identified splicing events in RNA were filtered out according to the following criteria:

1. number of junction carriers among non-cancer controls is >1;
2. number of junction carriers among patients is >3;
3. the number of patients, in which the number of events is supported by ≥3 junctions, is <1;
4. impact/function includes “annotated” or “present in another isoform”;
5. the number of junctions among patients <5.

Subsequently, prioritized events were classified pathogenic/likely pathogenic when fulfilling the following criteria:

1. filtered RNA events were matched based on the gene and chromosome coordinates to the filtered SNP DNA variants in each patient. Matched events/variants were inspected in IGV and evaluated whether the RNA event is aberrant and connected to the DNA variant. DNA variants leading to either outframe, or inframe containing functional domain, alternative 5’, or 3’ splice site, exon skipping or novel exon in at least 10 % of the present splicing events were considered pathogenic/likely pathogenic.
2. filtered RNA events present in ≥10 reads in patients and, at the same time, in junction fraction ≥0.2 in at least 1 patient and not present in non-cancer controls were inspected in IGV and evaluated whether the RNA event is aberrant and, if possible, connected to a DNA variant (SNP, or CNV). RNA events that were either outframe, or inframe containing functional domain, alternative 5’, or 3’ splice site, exon skipping or novel exon in at least 10 % of the present splicing events were considered pathogenic/likely pathogenic.

Prioritized RNA events caused by identified DNA variants (SNP or CNV) were added to the list of pathogenic/likely pathogenic variants used for further analyses (Supplementary Table S3). Altogether, 55 RNA events resulting in alternative 5’/3’ splice site, exon skipping, or novel exon formation were considered pathogenic/likely pathogenic. In 48 cases, the DNA variant causing aberrant splicing was identified including 22 variants close to the intron/exon boundary, but out of the canonical splice site (in the position ±3 up to 14 bp), 13 missense, or synonymous exonic variants, 8 deep intronic variants (further than 100 bp from the intron/exon boundary), and 5 CNV. In 7 cases, we were not able to identify the causal variant in DNA that led to formation of novel exon.

## PRS analysis

Raw NGS data were processed by an in‑house bioinformatics pipeline as described previously [1]. Using in-house scripts, a call rate PRS control was performed excluding samples or SNP with the call rate <95 % from the further analysis (Supplementary Table S5). The SNP sets were tested for HWE. Identity by State was performed in PLINK v1.90b6.26 to prevent duplicated samples. Missing genotypes were substituted with the observed mean value of the individual SNP across all probands as described previously by Borde at al. [12]. Treated VCF files were used for further PRS calculations. An individual’s PRS is calculated according to the following formula:

${PRS}_{i}=\sum_{i}^{N} {logOR}_{i}\times allelic dosage (0,1,2)$.

PRS represents the sum of log OR associated with the SNP multiplied by number of risk alleles (0, 1, 2) according to an individual’s genotype. For the calculation itself, we used the effect sizes of individual SNPs as published in the PGS Catalogue available online [13] or, if unavailable, from the respective studies [14-18]. The mean value of PRS in the PRS control group represents the standardized PRS value 0 (mean = 0, variation = 1). PRS value was standardized by comparison with PRS control group having mean 0 and variance 1. For any further calculations R v.4.2.0 was used. Differences between the standardized PRS values of patients and PRS controls were assessed using *t*-test with *p*-value less than 0.05 considered statistically significant.

## Supplementary references

1. Soukupova, J., et al., *Validation of CZECANCA (CZEch CAncer paNel for Clinical Application) for targeted NGS-based analysis of hereditary cancer syndromes.* PLoS One, 2018. **13**(4): p. e0195761.

2. Horackova, K., et al., *Low Frequency of Cancer-Predisposition Gene Mutations in Liver Transplant Candidates with Hepatocellular Carcinoma. LID - 10.3390/cancers15010201 [doi] LID - 201.* 2022(2072-6694 (Print)).

3. Walker, L.C., et al., *Comprehensive Assessment of BARD1 Messenger Ribonucleic Acid Splicing With Implications for Variant Classification.* Front Genet, 2019. **10**: p. 1139.

4. Hovhannisyan, M., et al., *Polygenic risk score (PRS) and its potential for breast cancer risk stratification.* Klin Onkol, 2023. **36**(3): p. 198-205.

5. Smit, A., Hubley, R & Green, P, *RepeatMasker software package*. 2013-2015.

6. Karczewski, K.J., et al., *The ExAC browser: displaying reference data information from over 60 000 exomes.* Nucleic Acids Res, 2017. **45**(D1): p. D840-D845.

7. Genomes Project, C., et al., *A global reference for human genetic variation.* Nature, 2015. **526**(7571): p. 68-74.

8. Exome Variant Server, N.G.E.S.P.E., Seattle, WA. Available from: <http://evs.gs.washington.edu/EVS/>.

9. Landrum, M.J., et al., *ClinVar: improving access to variant interpretations and supporting evidence.* Nucleic Acids Res, 2018. **46**(D1): p. D1062-D1067.

10. Leman, R., et al., *SPiP: Splicing Prediction Pipeline, a machine learning tool for massive detection of exonic and intronic variant effects on mRNA splicing.* Hum Mutat, 2022. **43**(12): p. 2308-2323.

11. Jaganathan, K., et al., *Predicting Splicing from Primary Sequence with Deep Learning.* Cell, 2019. **176**(3): p. 535-548 e24.

12. Borde, J., et al., *Performance of Breast Cancer Polygenic Risk Scores in 760 Female CHEK2 Germline Mutation Carriers.* J Natl Cancer Inst, 2021. **113**(7): p. 893-899.

13. Lambert, S.A., et al., *The Polygenic Score Catalog as an open database for reproducibility and systematic evaluation.* Nature Genetics, 2021. **53**(4): p. 420-425.

14. Bolton, K.L., et al., *Common variants at 19p13 are associated with susceptibility to ovarian cancer.* Nat Genet, 2010. **42**(10): p. 880-4.

15. Pharoah, P.D., et al., *GWAS meta-analysis and replication identifies three new susceptibility loci for ovarian cancer.* Nat Genet, 2013. **45**(4): p. 362-70, 370e1-2.

16. Goode, E.L., et al., *A genome-wide association study identifies susceptibility loci for ovarian cancer at 2q31 and 8q24.* Nat Genet, 2010. **42**(10): p. 874-9.

17. Phelan, C.M., et al., *Identification of 12 new susceptibility loci for different histotypes of epithelial ovarian cancer.* Nat Genet, 2017. **49**(5): p. 680-691.

18. Jervis, S., et al., *A risk prediction algorithm for ovarian cancer incorporating BRCA1, BRCA2, common alleles and other familial effects.* J Med Genet, 2015. **52**(7): p. 465-75.
